# Developing a Digital Tool to Calculate Protein Quality in Plant-Based Meals of Older Adults: User Engagement Design Approach With End Users

**DOI:** 10.2196/48323

**Published:** 2024-12-19

**Authors:** Lotte van Dam, Sine Højlund Christensen, Inge Tetens, William Riley III, Mariëlle Timmer, George Suciu Jr, Iuliana Marin, Lisette De Groot, Pol Grootswagers

**Affiliations:** 1 Division of Human Nutrition and Health Wageningen University Wageningen Netherlands; 2 Department of Nutrition, Exercise and Sports University of Copenhagen Copenhagen Denmark; 3 Wageningen Food and Biobased Research Wageningen University & Research Wageningen Netherlands; 4 Research & Development Department, BEIA Bucharest Romania; 5 Faculty of Engineering in Foreign Languages University Politehnica of Bucharest Bucharest Romania

**Keywords:** digital tool, protein quality, user engagement design approach, plant-based diets, healthy ageing, mobile phone

## Abstract

**Background:**

The global shift toward plant-based diets has been increasing, with more people making the transition for various reasons. In vulnerable subgroups such as older adults, the transition to plant-based diets deserves attention due to the potentially detrimental consequences of lower protein quantity and quality.

**Objective:**

We aimed to develop a digital tool that ensures adequate protein quality in plant-based meals for older adults experiencing low protein intake through an interdisciplinary collaboration and user engagement with potential end users.

**Methods:**

Three focus group interviews of Dutch and Danish dietitians and older adults as potential end users were conducted to identify their needs, preferences, and deal-breakers. Focus group interviews were based on a user-task-environment analysis, the Walt Disney method, the brainwriting method, and a cognitive walkthrough. The interview transcripts were analyzed with a thematic analysis. The front end and backend development of a potential tool took place in parallel and was well-synced to the focus group interviews.

**Results:**

Both dietitians and older adults from Dutch and Danish sites expressed high interest in a tool that provides feedback and background information on protein quality, sustainability, and nutrients or micronutrients. The user-task-environment analysis delivered input among others that dietitians and older adults are good potential users, the tool should be functional as an app as well as a website and the tool should provide preprogrammed meals or recipes. The Walt Disney method delivered usable and realistic solutions to the 4 challenges presented. Thirty-two percent of the solutions on all themes presented with the brainwriting method appeared to be highly feasible and relevant, having the potential to be implemented in a tool. The cognitive walkthrough identified certain screens as unclear, necessitating revisions for improved understandability, for example, the need for explanation in selecting food item filters is shown in screenshot 2, with an overall usability score of 59%.

**Conclusions:**

Our user engagement design approach resulted in a prototype that ensured end users’ wishes and needs, with a finetuned output tested in focus groups. We conclude that our user engagement design approach was a suitable and meaningful stepwise approach to ensure the relevance of the tool and identify potential barriers. The focus group results indicate that dietitians have a clear understanding and need for a tool to aid in meal planning for enhanced protein quality, highlighting its absence in their current resources despite increasing demands arising from the protein transition. Conversely, for older adults, the introduction of a digital tool appears less appropriate; instead, there is a necessity for foundational education on protein quality before such a tool can be effectively used. Future studies are needed to further implement the prototype into practice.

## Introduction

Societal trends and governmental pressures steer populations toward more plant-based diets due to their environmental benefits and positive health outcomes, such as reduced risk of heart disease and certain cancers [1-4]. These diets are gaining popularity due to growing awareness of their benefits, alongside policy initiatives aimed at promoting sustainable eating habits. However, the transition to plant-based diets must consider the specific nutritional needs of different population groups, because plant-based food items have a lower essential amino acids (EAA) content compared to animal-based foods [5-7]. A successful protein transition should also benefit the growing population of older adults (ie, 65 years and above). Currently, 106 million older adults in Europe comprise 21% of the total population, a proportion expected to rise to 30%, or 150 million, over the next 3 decades [8]. Older adults who consume traditional diets but who have less appetite than earlier are already at risk of insufficient protein, vitamin D, and vitamin B12, nutrients, all of which are typically derived from animal sources [9-11]. As vitamin D can also be synthesized through sun exposure, limited time outdoors can exacerbate deficiencies, especially in older adults with restricted mobility or those living in northern climates. Thus, transitioning to plant-based diets in this age group must be carried out with care to avoid nutritional deficiencies that can lead to declines in muscle mass, bone health, cognitive functioning, and loss of independence [12].

Health authorities in the Netherlands recommend a 20% to 30% increase in protein intake for vegetarian and vegan diets to compensate for lower protein quality [13]. However, this strategy seems unsuitable for some older adults due to prevalent decreases in appetite [14,15] and the lower protein densities and higher fiber content of plant-based foods [16]. Moreover, increasing protein consumption to compensate for lower quality is counterintuitive considering the environmental goals of the protein transition. A more feasible strategy is careful meal planning, in which complementary protein sources are combined, to optimize the EAA composition. Yet, consumers often lack access to detailed information on amino acid content, digestibility, and recommendations, making meal planning complex.

While identifying complementary plant-based protein sources is challenging for consumers, digital tools can streamline this process. Using databases that contain information on EAA content, algorithms can be set up to identify optimal food combinations to meet nutrient requirements. Such tools can aid those seeking to eat more sustainably and concurrently, prevent nutritional deficiencies. Existing nutritional assessment tools such as Optimeal [17] and Fortifull [18] provide general guidance but do not incorporate amino acid data crucial for evaluating protein quality.

To develop a useful and effective tool, 7 aspects should be addressed. First, the tool should rely on scientifically sound databases that have complete and accurate data on the amino acid composition and the digestibility of protein-containing foods. Second, the time window in which multiple foods can complement each other’s amino acid profiles needs to be defined. Third, personalized amino acid requirements per such a window should be developed [7]. Fourth, the tool must be trustworthy, ensuring that users can rely on its recommendations and data accuracy. Fifth, the tool should be functional and user-friendly, providing an intuitive and seamless experience. Sixth, it should be visually appealing and engaging to encourage regular use. Seventh, the development process should be user-centered, incorporating feedback and preferences from potential users to ensure it meets their needs and expectations.

In collaboration with nutrition scientists and food informatics experts, we aim to address these aspects of a digital tool. This paper describes the user engagement design approach with older adults and dietitians in Denmark and the Netherlands, investigating the wishes and needs for a tool ensuring adequate protein quality in plant-based meals for older adults experiencing low protein intake.

## Methods

### Methodological Approach

#### Overview

Given the exploratory nature of this study’s objective, a user engagement approach was chosen. Focus group interviews were applied to facilitate group interaction and enable the development of ideas through mutual elaboration [19]. This study is reported per the standards for reporting qualitative research [20].

#### Study Setting

This study was conducted in 2022 in parallel at the University of Wageningen and the University of Copenhagen. This study population consisted of older adults (>60 y of age) and dietitians who work with older people. The participant sampling was a purposive sampling.

#### Recruitment

The participants were recruited in both countries by reaching out through existing networks, social media, and mailing lists. In both countries, the dietitians were recruited throughout the whole country from multiple hospitals, private clinics, and municipalities. Older adults in both countries were recruited through established user panels and volunteer databases. Participants were eligible if they fulfilled the following inclusion criteria: older adults: ≥60 years of age, interested in plant-based diets or protein quality, familiar with smartphones or tablets, providing informed consent; and dietitians: working directly or indirectly and have experience with clients aged ≥60 years of age, providing informed consent.

#### Sampling Strategy

**Figure 1:**
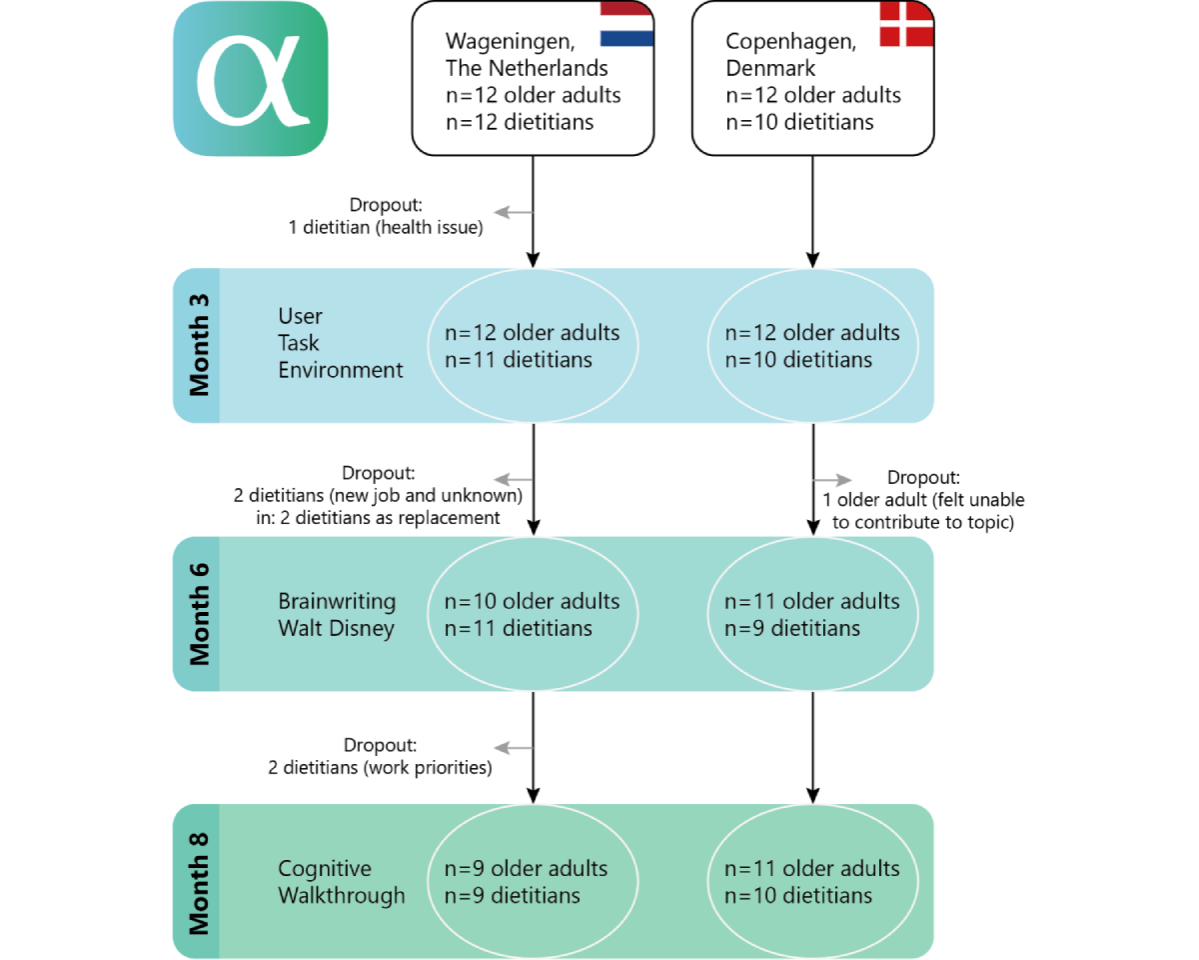
Flowchart of participants. In the Netherlands, two dietitians were not present due to work priorities during the cognitive walkthrough, two older adults were not present during the second focus group, one due to vacation and one due to surgery. During the third and final focus group, three older adults were not present due to other priorities, and one due to health issues. In Denmark, one dietitian missed the second focus group due to illness.

The goal was to reach a total of at least 20 older adults and 20 dietitians. In total, 22 dietitians and 24 older adults participated in this study (Figure 1). Focus group meetings were held with 4 to 7 participants per group. The participants were invited to attend all 3 focus group interviews. Except for 2 married couples in the Netherlands and 1 in Denmark, none of the participants knew each other beforehand. The composition of the 3 focus groups’ meetings differed throughout the 3 sessions due to the participants’ availability.

### Ethical Considerations

This study was approved by the local university Research Ethics Committees in both countries (Denmark: Ethical Committee of SCIENCE and SUND, University of Copenhagen: 504-0325/22-5000, Netherlands: Social Sciences Ethical Committee: 2022-50-Grootswagers).

### Description of the Methods

#### Overview

Focus group interviews were held during three phases: (1) understanding (need assessment), (2) conceptualizing (development and refinement to meet specific wishes), and (3) testing, with all participants going through all 3 phases. The focus group structure was standardized across the two countries by using the same script, questions, and agenda, all developed in English. The sessions were facilitated by trained researchers from each country, serving as moderators. These moderators tried to allow every participant to share their opinions in discussions, asked for further elaborations, and tried to create an open and safe space to speak their minds. The focus groups’ sessions with the dietitians had a duration of 2 hours per session, while the focus groups with older adults had a duration of 3 hours per session. These durations were chosen to account for an estimated higher need for explanations and more elaborate answers with older adults.

**Figure 2:**
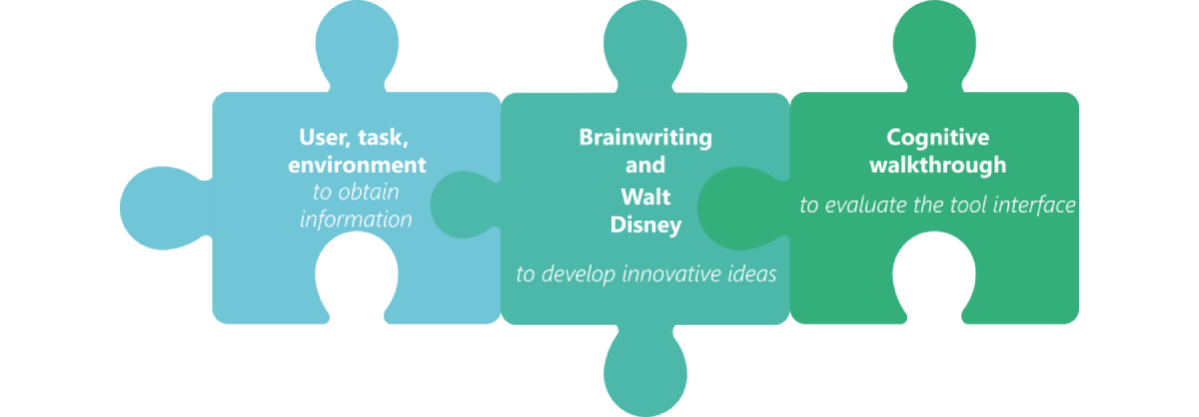
The goals of each of the three phases in the development of a digital tool that can ensure sufficient protein for older adults.

All focus group interviews were held in June, September, and November 2022, all scheduled in the afternoon. The 3 phases for the focus group interviews were constructed in Figure 2.

A user-task-environment analysis [16] was conducted during the first interview session to understand the needs of end users. In this session, data were gathered on the user (user), the relevant functionalities of the tool (task), and the intended usage environment (environment). Questions about these 3 topics were combined into a script (Multimedia Appendix 1). During the focus groups, the script was used as a guideline, while participants were free to talk, and questions were asked to elaborate further on what was said. Sound recordings were made of all focus groups in duplicate, using either mobile phones or an audio recorder (Philips Voice Tracker DVT6110). These sound recordings were used for further analysis.

In the conceptualizing phase, the second interview session used 7 specific questions, applying the Walt Disney (WD) method [21] and the brainwriting method to develop innovative ideas [22]. In the WD method, 4 specific questions or problems were presented, and the participants were divided into 3 different roles to generate ideas and state barriers: dreamers, realists, and critics as described by Dilts et al [23] and Dilts [21]. Sound recordings during the WD method were made for further analysis. In the brainwriting method, which is a validated method for high-quality idea generation [22], 3 specific questions or problems were presented to the participants. Every participant wrote down 3 suggestions for solving the question or problem (3-6 min). The forms were then passed on to the next participant who refined or amended the suggested ideas until everyone had their original form back. These forms were used for further analysis. For both the WD method and the brainwriting method the minimum required outcome was the provision of at least one idea or suggestion for each question presented.

In the testing phase, the third interview session was conducted as a cognitive walkthrough (CW). Here, the participants were shown 8 static screenshots of the future tool and asked for feedback [24]. The screenshots were shown without additional explanation, except for necessary translations into the native language. The three specific questions that were asked about the screenshots were the following: (1) Do you know how to navigate on this screen? (2) Do you understand everything on this page? (3) Do you find the function relevant? Participants answered “yes” or “no,” and in the case of a “no,” a brief discussion was held about the answers. After every screenshot, a short discussion was held about their written answers. The written forms, including all the answers and the notes made during the discussions afterward, were used for further analysis.

#### Development of the Databases and Algorithms

The database used for the first development steps of the tool was based on the NEVO (Dutch nutritional database, Nederlands Voedingsstoffenbestand)–table, which is the Dutch national food database that contains unique codes for ~2500 food items and information on nutrient composition. In a separate project by the Division of Human Nutrition of Wageningen University, a database was built that linked digestibility factors and amino acid contents to NEVO codes. The full description of the methodology can be found elsewhere [25]. In short, digestibility factors were added by averaging known protein digestibility corrected amino acid score values of foods within a food group to a joint correction factor, weighted by the relative frequency in which food items within a category are consumed. Amino acid contents were added to all foods containing ≥1 EN% protein. Amino acid data were used from the national food databases of Denmark, the United States, the United Kingdom, and Japan, in that order of priority based on data availability and cultural similarity. If products did not have a similar direct equivalent, 4 different solutions were considered: (1) the amino acid data of the source protein was used if the proteins in the food item mainly originated from 1 source, (2) amino acid data of highly comparable products were used, or (3) a recipe was built based upon the relative content of different protein sources with known amino acid contents. In the case of database differences in total protein content, (4) amino acid data were scaled to the protein content reported in NEVO.

**Figure 3:**
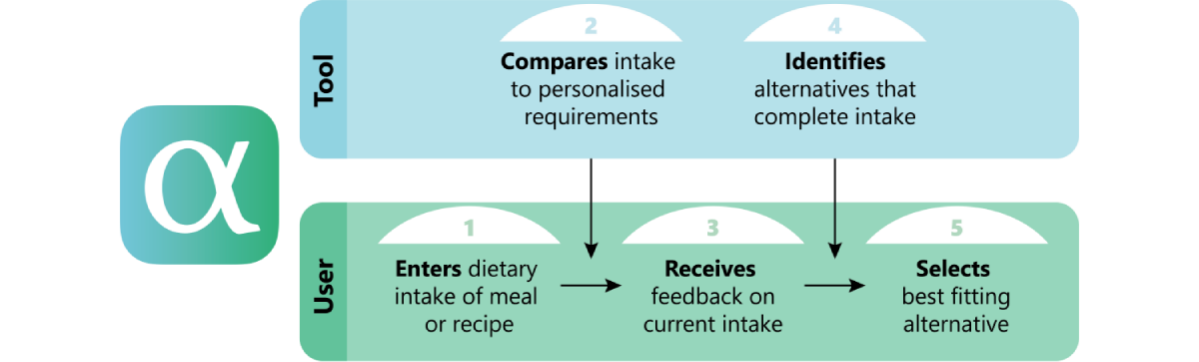
Flow of the proposed solution.

For step 2 in the workflow (Figure 3), personalized requirements for EAAs needed to be developed. The detailed overview of the development and validation of these requirements is beyond the scope of this paper. In short, for the 3 main meals of a day threshold values for all EAAs were established, from the perspective of optimal muscle protein synthesis in older adults. The extent to which the current meal contributed to the threshold values for the EAAs was expressed as a Meal Protein Quality Score (MPQS) and fed back to the user (step 3). MPQS ranges from 0 to 100, where 0 means that 1 EAA is completely absent in the meal and 100 means that all EAAs are consumed above the threshold values. In step 4, alternatives, such as a change or an addition to the meal, are identified.

We considered 3 different algorithms designed by experts in nutrition and food informatics:

- Gram-by-gram alternative: for a specific protein-containing item in the meal, a simulation is performed in which the exact consumed amount of this food item (in grams) is replaced by a similar food item, but all other food items in the meal are kept fixed (ie, no alternatives are proposed for them). Food item similarity is based on taste, food category, and empirical evidence for frequent coconsumption with the other meal items. For every potential replacement with the alternative, a new MPQS is calculated for the meal (keeping the other food items fixed), and the alternatives that lead to the largest improvement in MPQS are presented as optional alternatives.
- Missing-piece alternative: the difference in the required amino acid profile and consumed amino acid profile is used to find any NEVO item that will increase the MPQS to 100 once added to the meal. The alternatives are sorted in ascending order by the amount of grams that would need to be consumed of the item.
- Proportion-adjustment: by linear programming, the influence on MPQS of adjusting the proportions of food items within the meal is determined, and the best ratio is presented as an alternative. The constraints are that the total weight of the meal cannot be adjusted and that the adjusted intake of the food items stays within 50% to 200% of the current intake.

### Data Analysis

For the analysis of the data from the first focus group (user-task-environment analysis) and the analysis of the data from the WD method in the second focus group, sound recordings were transcribed manually, in their native language. A thematic analysis was used [19]. An English coding book was made based on the script. This was used for coding the transcription in both sites (for the full coding book, see Multimedia Appendix 2). Examples of codes used were crucial functions, interesting thoughts, doubts, and deal-breakers. Additional codes that were made during analysis were added to the coding book, so the codes were consistently used in both sites. Coding of the transcriptions was carried out using the program NVivo (QSR International, release 1.6.1 {1137} and release 1.7 {1533} in both sites, using English codes. A cross-check was performed by the two researchers performing the coding, with a short part (~10%) of both transcriptions translated into English to keep consistency. Where differences occurred, the codes were discussed until full agreement was reached. After coding, selected quotes were sorted, translated into English, and then combined from both sites. Duplicates were removed, with a note behind it, showing that multiple participants agreed upon it (both within and between sites with a separate notation). The combined results were presented to the tool development team.

For the analysis of the brainwriting method of the second focus group, results were translated, sorted, combined, and scored to develop a selection list and were scored based on 3 factors: developmental feasibility, scientific feasibility, and scientific relevance, including nutrition, health, and sustainability. The 3 factors were scored individually by 2 professionals recruited within the project team with expertise in the respective areas. One could score a 1 when not feasible or not relevant, and a 3 if easily feasible or very relevant. The end score was determined by the lowest score. If needed, comments were added to the scoring list. Based on this scoring list, important changes to the tool were identified and presented to the tool development team.

The results of the third focus group (CW) of the participants’ understanding of the screenshots were quantified. The CW consisted of 3 questions for each of the screenshots, related to (1) navigating, (2) understanding, and (3) relevance. The frequency of “yes” (“yes I do know how to navigate/understand/see the relevance”) and “no” was counted and percentages were calculated. When both a “yes” and “no” were encircled, this was scored as a half yes or no, and when nothing was encircled, this was considered as missing data. Suggestions and further explanations from the participants were all sorted, translated, and combined from both sites. The frequency of how often the same suggestions were given by the participants was counted and these suggestions were placed above, in a ranking order. This ranking order was presented to the tool development team.

## Results

### Overview

In this study, we recruited 22 dietitians and 24 older adults.

### Baseline Characteristics

Of the total of 22 dieticians, all were female in both countries, whereas with the older adults (n=24), a total of 50% (n=6) were male in the Netherlands and 25% (n=3) were male in Denmark (Table 1). The age range of the dietitians was 26-60 years in Denmark and 26-64 years in the Netherlands, and the age range of the older adults was 68-79 and 62-89 years, respectively, in Denmark and the Netherlands. With regards to the workplace of dietitians, in both countries, various parts of the country were represented (data not shown). In both countries, most of the older adults were used to cooking for themselves. All older adults participating in the focus groups were interested in eating plant-based diets.

**Table 1:** Characteristics of the focus group participants.

		Netherlands	Denmark
**Dietitians^a^**			
	Sex (male/female), n/n	0/12	0/10
	Age (y), median (range)	32 (26-64)	37 (26-60)
	Working place (hospital/municipality/private), n/n/n	2/4/6	6/4/0
	Work experience (years), mean (SD)	17.8 (15.6)	12.9 (14.4)
**Older adults^b^**			
	Sex (male/female), n/n	6/6	3/9
	Age (y), median (range)	67 (62-89)	75 (68-79)
	Education (high/intermediate/low), n/n/n	5/7/0	11/1/0
	Living situation (cohabiting/alone), n/n	10/2	4/8
	Cooking habits (never/sometimes/always), n/n/n	2/3/7	0/0/12
	Dietary habits (omnivore/flexitarian/vegetarian), n/n/n	5/6/1	12/0/0

^a^Netherlands: n=12 and Denmark: n=10.

^b^Netherlands: n=12 and Denmark: n=12.

### Understanding

#### Identifying User Needs With the User-Task-Environment Analysis

According to all participants, the tool might be interesting for diverse target populations. Examples were young families, pregnant women, patients undergoing surgery, athletes, older adults, health care practitioners, and people interested in eating vegan or vegetarian food.

#### Visual Aspects and Format

Big fonts and good contrast were important for all participants. Pictures were highly valued in both target groups, for example, images of products or alternatives and the use of icons that are already familiar.

Dietitians and older adults wanted either a smartphone app or a website, with a preference for a website for dietitians, and a preference for a smartphone app for older adults. For both target groups at both sites, security was important or necessary. A possibility should be present to use the tool without the login step.

#### Nutritional Input and Feedback

Dietitians wanted to fill in the nutritional intake during consultations, together with the client. Both target groups wanted the possibility to fill in personal information (such as age, sex, protein requirement, allergies, and food preferences). With entering the dietary intake, dietitians wanted to be able to enter the basic ingredients. When their clients have to enter the data, the possibility to work with pictures of food would be valued, which was in agreement with the preferences of the older adults. For both target groups, it was important that the dietary assessment be easy and quick. The tool should be able to remember input history, should be flexible in the amount of the product (user can choose between spoon size, portion size, or amount in grams), and should be short (just for 1 meal).

Both target groups wanted to receive feedback per meal moment and an overall conclusion of the intake over the whole day. Both target groups wanted feedback on protein quality (amino acids), micro- and macronutrients, and sustainability. Visually, it should be immediately clear if the intake does not meet requirements. Dietitians wanted the feedback to be clear and rather harsh, while the older adults wanted to receive feedback in a positive manner.

Dietitians wanted to obtain between 3 and 5 alternatives presented by the tool, while older adults prefer to receive between 3 and 10 alternatives. Both target groups wanted to have the alternatives shown in a ranking with the best alternative shown at the top, including information on the reasoning behind the alternatives, and the amounts of the alternative products that should be consumed. Alternatives should be presented based on personal preference, culture, season, location of origin, and price.

In general, the tool should provide background information. “Motivation often comes from knowledge” according to one of the dietitians. Therefore, both target groups wanted short texts and short videos. Background information should contain information on the general idea of the application, amino acids, muscle mass, recipes, the difference between plant-based and animal-based protein, and sustainability.

#### Crucial Functions, Interesting Thoughts, Doubts, and Deal-Breakers

According to all participants, the tool should trigger the user’s interest and should function easily and quickly, with the fewer clicks the better. Both target groups preferred integrations or API (application program interface)–connections with other dietary intake tools. Feedback should be savable to allow for retrieval at a later stage and for the dietitians exportable in PDF format for communicative and administrative purposes. The tool should be updated regularly to include the newest food item data. Dietitians wanted to be able to change the protein requirements for their clients themselves.

Other additional thoughts from both target groups at both sites were that the tool provides preprogrammed meals or recipes. For older adults, it might be motivational if there was a community of users with whom experiences and recipes can be shared. Both target groups request the possibility to give feedback to the software developers.

Dietitians had some doubts about the tool, especially about the number of plant-based products necessary to consume to reach amino acid requirements in case of decreased appetite. Other doubts of dietitians at both sites were that clients lack motivation due to a lack of knowledge and that clients are digitally incapable of using such a tool. Older adults had some doubts about the tool, especially about the time and energy needed to fill in nutritional information, and about the suitability of the tool for older users.

For both target groups, deal-breakers were the lack of speed of the tool, too much time investment needed, unsuitable alternatives presented, and complexity of the tool. A deal-breaker specifically for dietitians was missing products in the database. A deal-breaker for older adults was the price of the tool.

#### Environment

The environment in which the tool might be used according to the older adults at both sites is especially at home, and some also wanted to bring the tool to the supermarket. Dietitians wanted to use the tool together with their client during consultations, being able to immediately show the feedback to the client.

At this step, 1 target group had to be identified to tailor the development of the back end and the front end. The consortium agreed to focus on dietitians for the first development. This was due to the complexity of the topic. Additionally, it was seen as an advantage to have experts in nutrition as the first target group in the development of the tool, as that will likely result in receiving more valuable feedback on how to improve the tool and reduce the risk of misinterpreted information.

### Conceptualizing

#### Developing Solutions With the WD Method

The themes discussed in the WD method were the following: (1) What to do with consumed food items that are not present in official nutritional databases in the Netherlands and Denmark (NEVO and Frida)? (2) How can we explain the relevance of receiving feedback and further background information? (3) How do we keep data entry exciting? (4) Which results are essential to export? (dietitians only).

According to both target groups at both sites, the best solution when food items are not present in the databases was to fill in a tick-box indicating that this is feedback for the software development team. In this way, the missing product information is fed back and can be added with an update. Another solution was to have other comparable products presented immediately. Other solutions were that the users search for a comparable product or that the user would upload a picture of the food item so the tool could come up with comparable products.

Several solutions were brought up by dietitians on how to explain the relevance of the tool, such as information icons to click on, knowledge clips, the possibility to click through for further information, newsletters, and a community of users involved in development. The older adults suggested knowledge clips, informative cartoons, famous ambassadors, and providing information in speech.

Dietitians provided solutions for how to keep data entry exciting, such as suggesting previously consumed products and meals, and the ability to ignore small typographical errors. Older adults looked at the data entry from a different perspective and came up with solutions, such as data entry in the form of a game, using sounds, and traffic lights. Suggesting previously consumed products and meals was also stated by the older adults in both countries.

Lastly, for the fourth question, dietitians at both sites reported that it is essential for the results to include the original intake of all amino acids relative to the requirements. They also wanted the possibility to choose other nutrients (macro and micro) on which they could receive feedback.

#### Developing Solutions With Brainwriting Method

Categories discussed with the brainwriting method were: How should feedback look like for (1) protein quality, (2) other nutrients, and (3) sustainability? (4) Which criteria would you desire to filter alternatives? (5) What do you want to obtain from the tool?

Suggestions were scored on 3 criteria: development feasibility, scientific feasibility, and scientific relevance from 1 to 3, where 3 indicates high feasibility or relevance (Multimedia Appendix 3). The total score was based on the lowest score.

For all categories, suggestions with high feasibility and relevance were provided. Of all the suggestions obtained 23 of 72 suggestions had a total score of 3, which is 32% of the total amount (52% scored a 2 [37/72], and 17% scored a 1 [12/72]). Of the feasible and relevant suggestions, 39% (9/23) were about protein quality. Of the suggestions that were scored as 1 a total of 92% (11/12) were due to lack of scientific feasibility. It was judged that the data was not available at the moment (October 2022). This might change in the future. Find a detailed distribution of suggestions in Multimedia Appendix 3.

Among the highly feasible and relevant suggestions were the following: protein quality: Radar chart of EAAs with circles indicating 100%, and then details can be seen when the “cursor” is placed upon it; other nutrients: Use of household measures; and sustainability: a suggestion for more information with a click on an I-icon. Regarding filters allergens, lactose, and animal proteins were among the suggestions that are feasible and relevant among all the assessment categories. Essentials of the tool sources to optimize protein quality with the highest content first, was a suggestion, which was found feasible for all 3 categories. The suggestions, which had a total score of 3 were used as direction for further development of the tool.

### Testing

#### Assessing Usability Using CW

**Figure 4:**
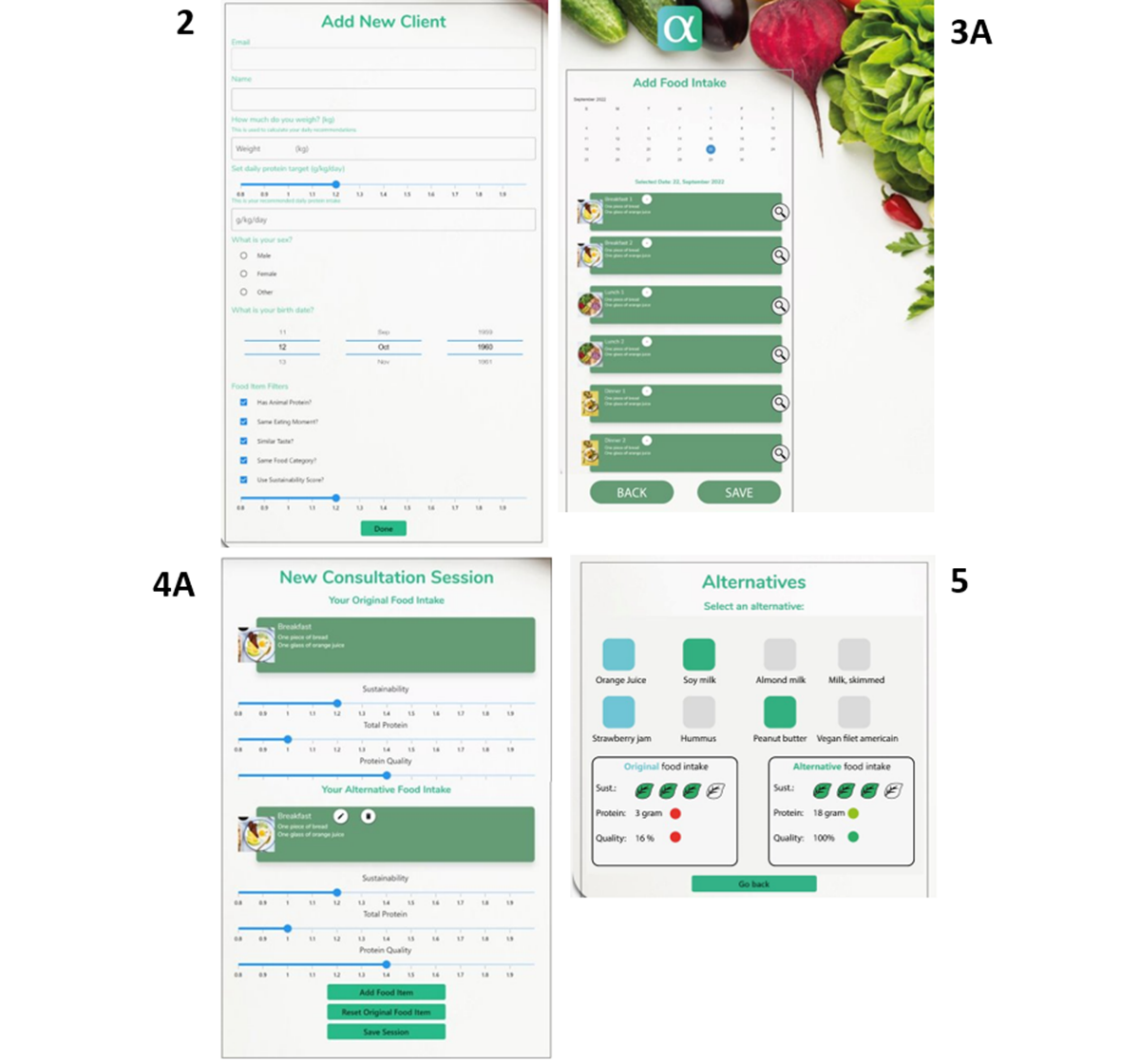
Screenshot 2: add new client, 3A: add food intake, 4A: new consultation session, and 5: alternatives; all used in the cognitive walkthrough. Screenshot 2 shows how to add a new client with client details; screenshot 3A shows how to add a new product separated by meal moments; screenshot 4A shows the feedback that is given on the original and alternative food intake on sustainability, protein intake, and protein quality; screenshot 5 is the screenshot where alternatives (green) can be chosen for the original products (blue).

In the CW screenshots were assessed by the user panel. Based on the average of the 3 questions asked per screenshot, the 4 most challenging screenshots were the same among the two target groups. The most challenging screenshots were screen 2: add new client, 3A: add food intake, 4A: new consultation session, and 5: alternatives, compare Figure 4 and Multimedia Appendix 4.

#### Screenshot 2: Add New Client

Screenshot 2 has an overall usability score of 59% among the dietitians, driven by understanding (4/19, 21%), relevance (14/19, 74%), and navigation (15.5/19, 82%; Figure 3). The understanding was lacking at both sites due to a need for an explanation of the specific food item filters, and no meaning of the unit of the sustainability bar.

Screenshot 2 had a usability score of 66% among older adults (Figure 3). The result showed that understanding had the lowest percentage (3.5/20, 19%), whereas relevance (14.5/18, 83%) and navigation scored higher (19/20, 95%). Both user panels suggested moving the filters to a later screen. As for dietitians, the challenge with understanding was due to food item filters (what do the different filters mean, and how many can a user choose?).

#### Screenshot 3A: Add Food Intake

The dietitians found it difficult to use screenshot 3A (46%), where the issues were mainly related to understanding and navigation (understanding: 1.5/19, 8%; navigation: 9/19, 47%; relevance: 15/19, 83%; Figure 3). The biggest challenges were related to the confusion about the calendar (why and the visual aspect of it) and the eating moments (how, where, and what to fill in). In addition, the meaning of the magnifying glass was not clear.

For the older adults, the screenshot that scored the lowest was 3A (60%) distributed among understanding (5/20, 28%), which was the lowest-scored category, followed by navigation (14.5/20, 74%) and relevance (15.5/20, 79%; Figure 3). As for the dietitians, the lack of understanding was due to confusion about the main meal headings regarding not knowing where to place drinks, snacks, and food eaten outside of main meals. Moreover, the meaning of the magnifier glass was unpredictable.

#### Screenshot 4A: New Consultation Session

Among all the screenshots, dietitians were mostly challenged by the usability of screenshot 4A (42%), both due to understanding (0.5/19, 3%) and navigation (9/19, 47%) of the screen (relevance: 14/18, 76%, compare Figure 3). The challenges with understanding were pointed out to be that it was not intuitive that the triangles would show more information, a lack of understanding of how the alternative food intake box functioned, and a lack of details. The challenges with navigation were pointed out to be not clear enough about what to do, and the heading was more misleading than guidance (Figure 3).

For older adults, the overall usability of screenshot 4A was 74%, with understanding as the lowest percentage (10/19, 51%), followed by relevance (14/17, 83%) and navigation (17/19, 89%; Figure 3). As for dietitians, the lack of understanding was due to not knowing the purpose of the triangles.

#### Screenshot 5: Alternatives

For screenshot 5 (57%), the dietitians scored understanding the lowest (6/19, 31%), followed by navigation (9.5/19, 50%) and relevance (16.5/18, 92%; Figure 3). The navigation was challenged by uncertainty about the meaning of the colors, and whether the alternative can be changed, whereas the lack of understanding was disturbed by ambiguity about whether the user has to choose something or not. Moreover, uncertainty about meal moments, the quantities of the alternatives, and whether the box shows the original food intake was present.

Screenshot 5 scored 64% for overall usability among older adults. Understanding had the lowest score (6/19, 36%), followed by navigation (12/19, 65%) and relevance (15/17, 90%; Figure 3). The understanding was disturbed by not understanding the color pattern and not knowing if the text on the screen was linked horizontally or vertically. Moreover, they expressed insecurity about how many food items the user can choose from this screen.

#### Similarities and Differences

The screenshots with the highest usability based on the average were the same among older adults and dietitians, in the same order of usability (the least difficult screen was 1 followed by 3C and 3B).

The biggest difference between older adults and dietitians regarding usability calculated by the average percentage of the 3 questions per screen was screenshot 4A with a 32% (74% versus 32%) difference and screenshot 3B with an 18% (87% versus 69%) difference, where the older adults had the highest score in both cases.

In general, the CW showed a need for higher consistency through screens, primarily due to visual aspects (colors or symbols), but also text (eg, save buttons).

## Discussion

### Principal Findings

In this study, we successfully used a user-centered design approach to assess the needs of a group of older adults and dieticians as potential end users for the digital tool under development. This study’s findings show that (1) the need for a tool was present among both target groups; (2) suggestions on how to handle nonpresent food items, how to explain the relevance of the feedback, and suggestions on how to keep the data entry interesting were all identified. For the dietitians suggestions for exporting data were also identified. (3) Feasible and relevant suggestions for the tool feedback were successfully identified per (a) protein quality, (b) other nutrients, (c) sustainability, (d) criteria for filtering suggested alternatives, and (e) desired benefit of use. (4) By testing the tools’ interface, challenging functions in the tool were identified. This approach allowed us to both develop and refine various solutions for the tool. However, during the development and refinement phase, differences in the desired solutions between the two groups became apparent. Due to resource constraints, we decided to focus on developing the prototype for 1 target group only, ultimately choosing dietitians for three reasons: (1) the physiological and nutritional understanding that is needed to successfully operate the tool is present in dietitians but would have to be trained in older adults; (2) to avoid the risk of misconception, the tool can deliver raw feedback, which the dietitian hands over to older adults in relevant context; and (3) dietitians are skilled in data entry and will be better able to find food-item alternatives in the occasions where products are missing in the dataset because of their professional experience and knowledge. The dietitians involved in this research have presented their support and interest in the tool until the end of data collection, and many dietitians have expressed their interest in being involved in its further development, indicating that the tool does fill an existing demand among dietitians.

During the development phase of the digital tool, the main landmarks that were achieved were: (1) the development of databases with data on nutritional content, amino acids, digestibility, and sustainability and (2) the development of algorithms that fetch nutritional data from the database and that calculate MPQSs*.* The development of the algorithms that identify alternatives has been commenced and needs further development. Moreover, the assumptions underlying the calculations of MPQS need validation, and the functionality of the feedback-providing algorithms should be tested on real-life data. These tests ideally comprise quantitative testing, where improvements in MPQSs over time are visible after the implementation of presented alternatives, and qualitative testing, where presented alternatives are scored on cultural, culinary, and nutritional relevance. Moreover, the tool has been developed based on the Dutch NEVO food database. To ultimately implement the tool worldwide, more food databases should be supplemented with data on amino acids and digestibility, and algorithms should be easily convertible to other food databases.

### Comparison With Prior Work

In this study, we had a 3-phase user-centered design approach. This 3-phase approach allows us to understand the user’s needs per different functionalities. Maramba et al [26], evaluated the use of usability of a testing phase in 133 eHealth tools (including nutritional tools) in a scoping review and found that less than 1/3 of the tools did undergo usability testing and that the use of “think-aloud” resulted in at least 1 further relevant iteration. Further iteration supports the relevance of approaching the development using methods that allow end users to have high involvement in the idea-generation process.

Other recent studies have used a similar approach compared to this study, with a high focus on end users and usability testing in the development of health-related tools [27,28], some of which are nutritional tools [27,28]. The development of the tool Dieta Dash (Alebg) had the purpose of giving the best food choices for preventing and evaluating hypertension [27]. The target audience sample (primary care physicians and nutritionists) was asked questions on how the prototype of the tool could be improved. They found that quick access to information, use of images, offline mode, and free access were some of the needs the end users had [27]. These needs are very similar to our results and strengthen the idea that this is a universal need for end users when they use eHealth tools. Kavanagh et al [28] developed a web-based health app (PortFolioDiet) for cardiovascular risk reduction. Here they used a 2-phase usability testing, including the acceptability of the tool. They found that a user guide on how to navigate the tool could be useful. This result was also seen in our study, and this strengthens the likelihood that it is a crucial function to include in a tool.

Loureiro et al [29] used focus group interviews in the development of a web-based tobacco tracker tool. The purpose of the focus groups was to explore ideas for the tracker prototypes’ content and design and also how to motivate people to use the tool, by using a thematic analysis of the data based on an a priori codebook and the development of new codes during the process. Although it is a completely different type of tool, the results were quite similar regarding user needs, where they found that positive reinforcement, gamification, and ease of use, for example, in the form of dropdown menus, are all relevant aspects [29].

Keniston et al [30] developed a tool for discharge planning. They also used a user-centered design strategy with several meetings with end users. According to the evaluation team, this approach, in close collaboration with end users, enabled the successful implementation of the tool in the hospitals. These findings support the probability that a tool that is developed in close collaboration with end users would be well-accepted.

The prototype of this tool had to be further developed, but based on the findings, we believe that the core functionalities have the potential to contribute positively to the target groups’ everyday lives by being a very practical and easy-to-use tool to raise awareness of protein quality and allow for the optimization of the individual’s diet concerning protein quality where relevant.

### Methodological Considerations

A strength of this study was the selection of participants. Specific inclusion and exclusion criteria ensured representative groups at both research sites, yielding targeted results. Consistent focus group interviews engaged participants deeply in the tool development, ensuring contextual understanding and relevant contributions. Recruited dietitians of diverse profiles, ages, and geographic backgrounds with experience in older adult nutrition enhanced suggestion validity. However, a limitation is potential selection bias. Dietitians who participated in this study might have been dietitians interested and supportive of protein transition. Thus, the findings may not be representative of all dietitians. The unequal gender representation in both focus groups could be seen as a limit, especially in the target group of older adults where the number of men is not representative of the gender distribution.

A further strength of this study is its use of focus group interviews. Contrary to individual interviews, a facilitated group discussion allows ideation through group interaction. This interaction ensured the ideas were created, discussed, and evaluated collectively, which has been seen to be a successful data collection process, also in the target group of older adults [31]. Moreover, such a discussion gives the individual a space to explore and rethink their point of view when meeting other perspectives. A risk of focus group interviews is that one participant might be dominant during a discussion compared to others. However, the moderators were aware of this risk and invited every participant to the discussion when relevant to reduce dominant respondent bias from the perspective of the moderators. Another strength of this study was the format and facilitation of the focus groups. Each focus group interview had a maximum of 7 participants which enabled everyone to become actively involved in the sessions [32]. Focus group interviews had a maximum duration of 3 hours for the older adults and 2 hours for the dietitians. While some older adults had difficulty focusing at the end of the session, we tried to maintain focus by holding breaks in between. This seemed to increase the focus and enable a more productive focus group.

During the first focus group interview, a short introduction round was held for participants to introduce themselves and their motivation for participation. Moderators asked follow-up questions to ensure participants felt heard. These initiatives may have increased the chance that participants felt safe to share their opinions openly. Moreover, in the first phase, moderators used open-ended questions and emphasized that there were no right or wrong answers. In addition, by asking follow-up questions, the moderators validated the statements of the participants when needed, which helped the moderators understand the statements, and thereby increased the validity of the data. Both moderators were neutral during the first focus group by not disrupting the discussion or leading it in a specific direction to reduce the risk of moderator bias.

Focus group interviews were conducted using a script and coding book. These steps increased consistency in data collection and data analysis across sites. Both research sites worked closely together in preparing, holding, and analyzing the focus groups. In the end, they compared the results and combined them. To avoid differences in coding between the two researchers at both sites cross-checks were performed by both researchers to check if coding was performed the same way. This also increased the reliability of the analysis.

By using the brainwriting and WD methods within the focus group interviews, we used a method of triangulation. This method combination increases the data quality by expanding and qualifying the understanding of the user’s perspectives and most important needs. The WD method not only made room for innovative ideas but also put the ideas into evaluation carried out by the role of critics. The critics contributed to an in-depth evaluation of the strengths and weaknesses of the ideas. The choice of the brainwriting method ensured concrete suggestions, increasing the chance that the implementation was aligned with the user´s needs. Brainwriting is used in similar research with success to include stakeholders in developing new patient programs, for example, in the contraception navigator program [33].

The stepwise increase in user involvement from the first until the last focus group interviews contributed to ensuring that a potential tool is developed in a way that fits the user’s needs. To have high user involvement when developing tools is used with success elsewhere [34].

A final limitation of the current approach is that by using a CW in the third phase as a way of testing usability we obtain quantitative results on the usability. These are difficult to compare with other usability studies. A way of improving this could have been by using the validated System Usability Scale as they give a comparable quantification of the results [35].

### Conclusion

Our user engagement design approach resulted in a prototype digital tool that ensured end users’ wishes and needs, with a finetuned output tested in focus groups. Our user engagement design approach appeared to be a suitable and meaningful stepwise approach to ensure the relevance of the tool and identify potential barriers. The prototype resulted in a finetuned output tested in the focus group sessions. The focus group results indicate that dietitians have a clear understanding and need for a tool to aid in meal planning for enhanced protein quality, highlighting its absence in their current resources despite increasing demands arising from the protein transition. Conversely, for older adults, the introduction of a digital tool appears less appropriate. Instead, before introducing such a tool, there is a necessity for foundational education on protein quality before such a tool can be effectively used. Future studies are needed to further implement the prototype into practice.

## Appendix

Multimedia Appendix 1 Script user-task-environment analysis.

Multimedia Appendix 2 Coding book.

Multimedia Appendix 3 Brainwriting method results table.

Multimedia Appendix 4 Cognitive walkthrough results.

## Abbreviations

API: application program interface
CW: cognitive walkthrough
EAA: essential amino acid
MPQS: Meal Protein Quality Score
NEVO: Dutch nutritional database, Nederlands Voedingsstoffenbestand
WD: Walt Disney
