# Supplementary material for: Developing a Digital Tool to Calculate Protein Quality in Plant-Based Meals of Older Adults: User Engagement Design Approach With End Users
Source: J Particip Med. 2024 Dec 19;16:e48323. doi: 10.2196/48323 (PMC11695958; doi:10.2196/48323)
Supplement: Multimedia Appendix 1 [file jopm_v16i1e48323_app1.docx]

Below follows the questions that formed the main part of the script for the first focus groups. Underlined questions were discussed, italic phrases were only used when needed. In the focus groups with the older adults, some introducing questions were asked before starting the UTE analysis:

- Which plant-based products (shown here) do you consume in your daily life? Why do you consume them or why not?
- Have you ever thought about changing to a more plant-based diet (eating more plant-based products and less animal based products)?
- Why do you want to change to a more plant-based diet – and why not?
- Since when did you want to change to a more plant-based diet?
- What did you change?
- Do you have any concerns about getting enough and the right proteins?
- Which factors influence your daily food intake?
- Do you have any experience with keeping track of your food/protein intake? (using a tool)?

User

For who can this be tool be interesting to use? What are characteristics of this user? Think of age, education, interest, health status etc.

Task

Suppose a tool can help you in switching to a more plant-based diet. What should the tool specifically do to help you?

What kind of tool would you like us to build? Think of an application, a website, a system of reminders that remind you what you can eat and when? Think broad! Also indicate how you would like this tool to function?

How should nutritional intake be entered?

- By product ingested?
- Would you like to be given a list of suggestions when entering your eaten products?
- Would you like to see a checkbox as a reminder to see if everything is completely entered?
- Who should enter the nutrition data? Would you do it yourself? The client or dietician?
- Would you like an option to indicate that you ate the same as the day before, per meal time?

How often do you want to use the tool? Preferably per meal 3 times a day, or enter all your consumed food throughout the day?

Suppose you enter your food intake and your intake is too low. What would you like to receive as feedback? What kind of information and in what form? (think of protein quality or amount of nutrients ingested or a sustainability score etc.)

How would you like to receive feedback after entering food intake? How should this feedback be visualized? *(Think of for example a slider that shows how close you are to the recommendation, or for example a traffic light system where you see a green, orange or red screen? Or just ‘eat less’, or ‘eat more’? think broad!)*

1. in what detail would you like this feedback?
   1. For example, think of a score with absolute numbers of your food intake, or just more visualization without concrete numbers,
   2. slider showing how close you are to the recommendation,
   3. traffic light system where you see a green, orange or red screen? And what would a red screen or a green screen indicate? Or does that not matter?
   4. Or just "eat less," or "eat more"?

What advice would you like to receive from the entered dietary intake? *(For example, think of a list of alternatives, a fixed list from which you can choose or 3 alternatives to add to your diet?)*

1. For example, think of a list of alternatives,
   1. how many would you like?
   2. Would you like to be able to indicate which products you would like to see alternatives of? Or does it not matter?
   3. And would you like to see a ranking of those as well, or just different alternatives?
   4. And how would you like the alternatives to be presented, just the name, a picture, a product description, a recipe?
   5. And would you like to know what these alternative products are based on? And if so, in what detail would you like that, an overall score, per meal, per product or on a protein/amino acid level)?

When and how often would you like to receive advice? (*Immediately or at the end of the day for example?)*

What should we, as tool developers, take into account in giving advice? *Consider cultural aspects, allergies, price of products, combinations with other foods, etc.?*

How can the user of the tool be encouraged to keep enter their nutritional data? How can we make the tool rewarding, and how can we prevent the user to become bored in the use of the tool?

How can the used be prevented from being afraid of giving ‘wrong’ answers?

Security of the tool (do you want to log in into the tool once or every time per use again?

Who should enter the nutritional data? Would you do that yourself?

Environment

When and where would you use the software? During or after diner? On computer, tablet or phone? Would you need your glasses, Wifi, phone access? Would you be able to use the tool when you are eating somewhere else? Always phone with you? Visibility?

Summary of the meeting and user questions

Would you use it? Why yes and why not?

Would you recommend it to other people? Yes to which people? No why not and what should be included in the tool so that you would use it?

Realistically, how many days per year (0 to 365 days) do you think you would actually use the software?

-Follow up question: What should be included in the tool to use it more often?

What do you think would motivate you to keep using the software? What would stop you from using the tool?
