# Supplementary material for: Developing a Digital Tool to Calculate Protein Quality in Plant-Based Meals of Older Adults: User Engagement Design Approach With End Users
Source: J Particip Med. 2024 Dec 19;16:e48323. doi: 10.2196/48323 (PMC11695958; doi:10.2196/48323)
Supplement: Multimedia Appendix 2 [file jopm_v16i1e48323_app2.docx]

Pre-defined codes (definition in brackets):

- Crucial functions (functions/aspects that should definitely be in the tool)
- User (for whom might the tool be interesting)
- Motivators plant-based (why participants want to eat more plant-based)
- Motivators animal-based (why participants want to eat animal-based)
- Motivators nutritional tool (why participants have used a nutritional tool before)
- Motivators tool (why participants want to use this tool or not)
- Input
  - Nutritional input (whether they want to fill in their nutritional intake)
    - Duration (time it takes to fill in your nutritional intake)
    - Amount (portion size/grams/spoons)
    - Frequency (how often they want to fill in their nutritional intake)
  - Individual input (whether they want to fill in their personal data, like age, weight, situation etc.)
- Feedback/output:
  - Feedback; Nutritional outcomes (what they want as feedback on their nutritional intake)
  - Feedback; Alternatives (What kind of alternatives do they want and how many?)
  - Feedback; frequency (How often do they want to receive feedback)
- Format (format of the tool, how it should function, basic stuff, like for example that the tool should be layered, with global stuff but also, if you want to, the possibility to go more in debt)
- Visual aspects
  - Visual aspects, general (how should the tool look like, in general? What are aspects we must keep in mind?)
  - Visual aspects, feedback (how should the feedback visually look like?)
- Deal breakers (what they do not want to have in the tool, otherwise they will not use it)
- Environment (where is the tool used, what are specifics)
- Security (what do they think about security aspects?)
- Doubts/uncertainties (factors that participants are still in doubt with)
- interesting opinions

Codes developed during analysis

- Demotivational factor (for example whether or not participants feel guilty about what they have to fill in about their nutritional intake)
- Feedback; background information (about the information that participants want, besides receiving feedback about their own intake)
- Experiences with a nutritional tool (earlier experiences)
- Responsible filling in information (who should fill in the information)

Dieticians:

Experience with clients and plant-protein (the experience of dieticians with clients and advising them about protein / plant-based protein)
