# Supplementary material for: Developing a Digital Tool to Calculate Protein Quality in Plant-Based Meals of Older Adults: User Engagement Design Approach With End Users
Source: J Particip Med. 2024 Dec 19;16:e48323. doi: 10.2196/48323 (PMC11695958; doi:10.2196/48323)
Supplement: Multimedia Appendix 3 [file jopm_v16i1e48323_app3.docx]

| Category |  | Development feasibility* | Scientific feasibility* | Scientific relevance | Total |
| --- | --- | --- | --- | --- | --- |
| PQ | Protein quality shown in percentages (= % of daily requirement) | 3 | 3 | 3 | 3 |
|  | Radar chart of essential amino acids with circle indicating 100%, and then details can be seen when the "cursor" is hovered over | 3 | 3 | 3 | 3 |
|  | Radar chart diagram: before-after to see the optimization. | 3 | 3 | 3 | 3 |
|  | Bar chart showing 100% filled with what is met for e.g., leucine | 3 | 3 | 3 | 3 |
|  | Red marking of limiting amino acids | 3 | 3 | 3 | 3 |
|  | Information on all amino acid | 3 | 3 | 3 | 3 |
|  | Thumbs up if the protein quality is good enough | 3 | 3 | 3 | 3 |
|  | The bars are coloured in red, yellow, green (green is sufficient nutrient intake - red is insufficient) | 3 | 3 | 3 | 3 |
|  | Colours indicating within (green) /outside (yellow) normal range of protein quality (no negative) | 3 | 3 | 3 | 3 |
|  | Red/yellow/green scale in relation to quality to show if level is sufficient - Visible at the following levels: daily diet - meal - food. | 3 | 2 | 3 | 2 |
|  | Scale/barometer showing how the user most easily reaches the target in terms of protein quality (both meal and food). | 3 | 2 | 3 | 2 |
|  | Pie cake diagram with total essential AA obtained - clicking unfolds it in more detail | 3 | 2 | 3 | 2 |
|  | Option to enter protein requirements yourself as a professional | 2 | 3 | 3 | 2 |
|  | Calculation of protein needs to provide suggestions for alternatives | 3 | 3 | 2 | 2 |
|  | Suggestions on optimization of the protein quality of the day/meal - amino acid level (in pictures and grams) | 2 | 2 | 3 | 2 |
|  | Suggestions on how much should be increased in terms of quantity to reach target | 2 | 3 | 3 | 2 |
|  | Suggestions for substitution rather than addition, in cases where the patient is a poor eater | 3 | 2 | 3 | 2 |
|  | Before and after guidance (substitution) so you can see the actual improvement, the user achieves, by following it. | 3 | 2 | 3 | 2 |
|  | Option to choose from different charts | 3 | 2 | 3 | 2 |
|  | Pictures of products and amino acids | 3 | 2 | 2 | 2 |
|  | Picture on how to solve the puzzle to make a complete protein out of amino acids | 2 | 2 | 3 | 2 |
|  | Example meals based on someone’s personal profile. | 2 | 2 | 3 | 2 |
|  | Show the different amino acids in different colors (helps to understand which amino acids are in different products) | 3 | 3 | 2 | 2 |
| ON | The bars can be folded out. Example: carbohydrate can be folded out and here details can be seen e.g. how much is fiber. (Click trough) | 3 | 3 | 3 | 3 |
|  | Mention the nutrients in grams and milligrams | 3 | 3 | 3 | 3 |
|  | Use household measurements | 3 | 3 | 3 | 3 |
|  | Macro nutrient energy content compared to the total amount of energy (kcal) of the meal | 3 | 3 | 3 | 3 |
|  | A box that can be "pulled out" by a click to see all nutrients | 3 | 2 | 3 | 2 |
|  | “NB”, marked with color, where extra attention is needed | 3 | 2 | 3 | 2 |
|  | Suggestions on where/how the user can get more or less of that nutrient. E.g. more fiber, less sugar | 2 | 2 | 3 | 2 |
|  | Option to leave out of nutrients that you are not interested in seeing feedback on | 2 | 3 | 3 | 2 |
|  | Examples for products in a menu | 2 | 3 | 2 | 2 |
|  | Information on the function of the nutrients for the body | 3 | 2 | 3 | 2 |
|  | Possibility to type in the different needs of the client to see if they are met | 1 | 2 | 3 | 1 |
|  | Possibility to type in the different needs of the elderly related disease specific nutrient needs (vitamin K / blood thinners, etc.) | 3 | 1 | 3 | 1 |
|  | The recommendation for respective nutrients should be shown | 3 | 1 | 3 | 1 |
| Su | Information with a click trough and an ‘i'-icon | 3 | 3 | 3 | 3 |
|  | Green leaves as sustainability score (One leaf is worst score and three leaves are best score) | 3 | 3 | 3 | 3 |
|  | Filter options as squares to select and deselect | 3 | 3 | 3 | 3 |
|  | Pictures of alternatives that are more sustainable | 3 | 2 | 2 | 2 |
|  | Knowledge of / suggestions for alternative options that make the meal more sustainable (Possibly first best choice that addresses amino acid profile or first best choice that addresses sustainability. Or both together) | 2 | 3 | 3 | 2 |
|  | Positive approach for sustainability | 3 | 3 | 2 | 2 |
|  | Option to go back to change filters | 2 | 3 | 3 | 2 |
|  | Ability to choose between different categories. (CO2, water use, packages use, animal welfare) | 2 | 1 | 3 | 1 |
|  | Feedback on sustainability related to the seasons | 2 | 1 | 3 | 1 |
|  | Local products as an alternative | 1 | 1 | 3 | 1 |
| Filt | Animal proteins as a filter | 3 | 3 | 3 | 3 |
|  | The system needs to remember the filters per account | 3 | 3 | 3 | 3 |
|  | Allergens as a filter | 2 | 2 | 3 | 2 |
|  | Lactose as a filter | 2 | 3 | 3 | 2 |
|  | Underweight as a filter --> should suggest high-calorie foods | 2 | 3 | 3 | 2 |
|  | Overweight as a filter --> should suggest low-calorie foods | 2 | 3 | 3 | 2 |
|  | Filter for high protein content (cut off value at 10 g / 100 g for example) | 2 | 3 | 3 | 2 |
|  | Filter for processed or natural foods | 2 | 2 | 3 | 2 |
|  | Price for the budget of the users (low - middle - high) | 2 | 2 | 3 | 2 |
|  | No added sugar as a filter | 2 | 1 | 2 | 1 |
|  | Food related to religious backgrounds as a filter | 2 | 1 | 3 | 1 |
|  | Foods that can better be avoided when diagnosed with a specific disease as a filter: (e.g. kidney disease: decrease salt; diabetes: decrease sugar) | 2 | 1 | 3 | 1 |
|  | Seasonal products as a filter | 2 | 1 | 3 | 1 |
| Obt | The tool should be easy to explore (so the professional can learn) | 3 | 3 | 3 | 3 |
|  | Visual effects like charts and pictures desired as output | 3 | 3 | 3 | 3 |
|  | Total energy% coverage by protein as output | 3 | 3 | 3 | 3 |
|  | Output as a PDF | 3 | 3 | 3 | 3 |
|  | An overview of proteins and amino acids as output of the tool | 3 | 2 | 3 | 2 |
|  | Suggestions for sources to optimize protein quality, highest content first | 2 | 3 | 3 | 2 |
|  | Explanation of amino acid composition desired as output | 3 | 2 | 3 | 2 |
|  | Ability to see changes over time in the output | 2 | 3 | 3 | 2 |
|  | Ability to export the output to the mail of dietician and patient | 2 | 3 | 3 | 2 |
|  | In the output a note function where you can attach comments to what is downloaded - sent to the patient which are comments / explanations to the guide / a personal message | 2 | 3 | 3 | 2 |
|  | A diary as output to be more aware of what is eaten | 2 | 3 | 3 | 2 |
|  | The tool can be used within a family with different people. Fill in all the wishes and see the overlap | 2 | 1 | 2 | 1 |
|  | Suggestions should be healthy, tasty and refreshing | 2 | 1 | 3 | 1 |

*Scoring of the Brainwriting results on feasibility (and to a lesser extent the relevance) might be depending on the actual state, and might change in ~10 years, when suggestions might become feasible.

PQ = Protein quality: How should feedback look like for protein quality; ON = Other Nutrients: How should feedback look like for other nutrients; Su = Sustainability: How should feedback look like for sustainability; Fil = Filters: Which criteria would you desire to filter alternatives; Obt = obtain: What do you want to obtain from the tool.

Scoring:

Development feasibility: 1=not at all feasible; 2=with lot of work; 3=easy to implement

Scientific feasibility: 1=data not available; 2=lot of work needed; 3=easy to implement

Scientific relevance: 1=irrelevant or not informative; 2=reasonable; 3=highly relevant
