# Supplementary material for: Developing a Digital Tool to Calculate Protein Quality in Plant-Based Meals of Older Adults: User Engagement Design Approach With End Users
Source: J Particip Med. 2024 Dec 19;16:e48323. doi: 10.2196/48323 (PMC11695958; doi:10.2196/48323)
Supplement: Multimedia Appendix 4 [file jopm_v16i1e48323_app4.docx]

Percentages of participants answering ‘yes’ to the questions.

Abbreviations: Navigation: Do you know how to navigate in this screen; Understanding: Do you understand everything on this page; Relevance: Do you find the function relevant; DK: Denmark, Copenhagen University; NL: The Netherlands, Wageningen University

Screen 1: Login


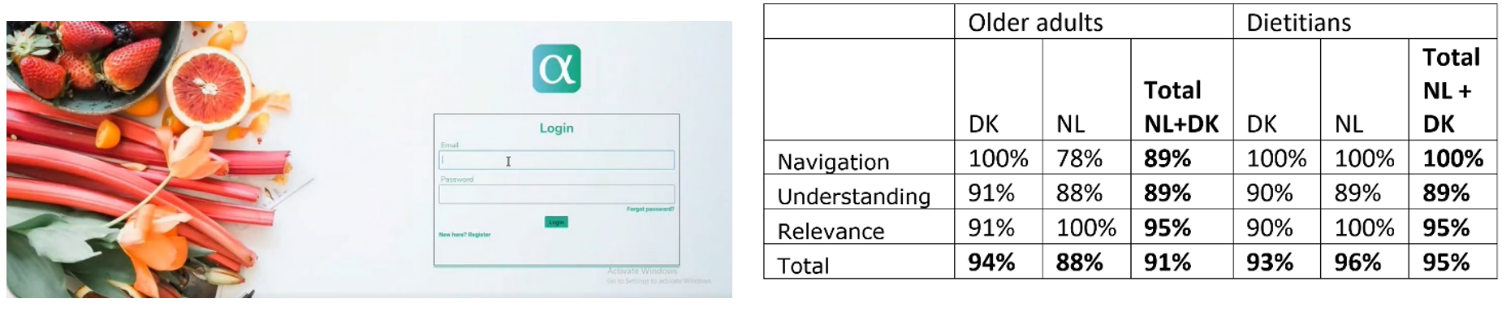


|  | Older adults | | | Dietitians | | |
| --- | --- | --- | --- | --- | --- | --- |
|  | DK | NL | **Total** | DK | NL | **Total** |
| Navigation | 100% | 78% | **89%** | 100% | 100% | **100%** |
| Understanding | 91% | 88% | **89%** | 90% | 89% | **89%** |
| Relevance | 91% | 100% | **95%** | 90% | 100% | **95%** |
| **Total** | **94%** | **88%** | **91%** | **93%** | **96%** | **95%** |

Screen 2: Add New Client


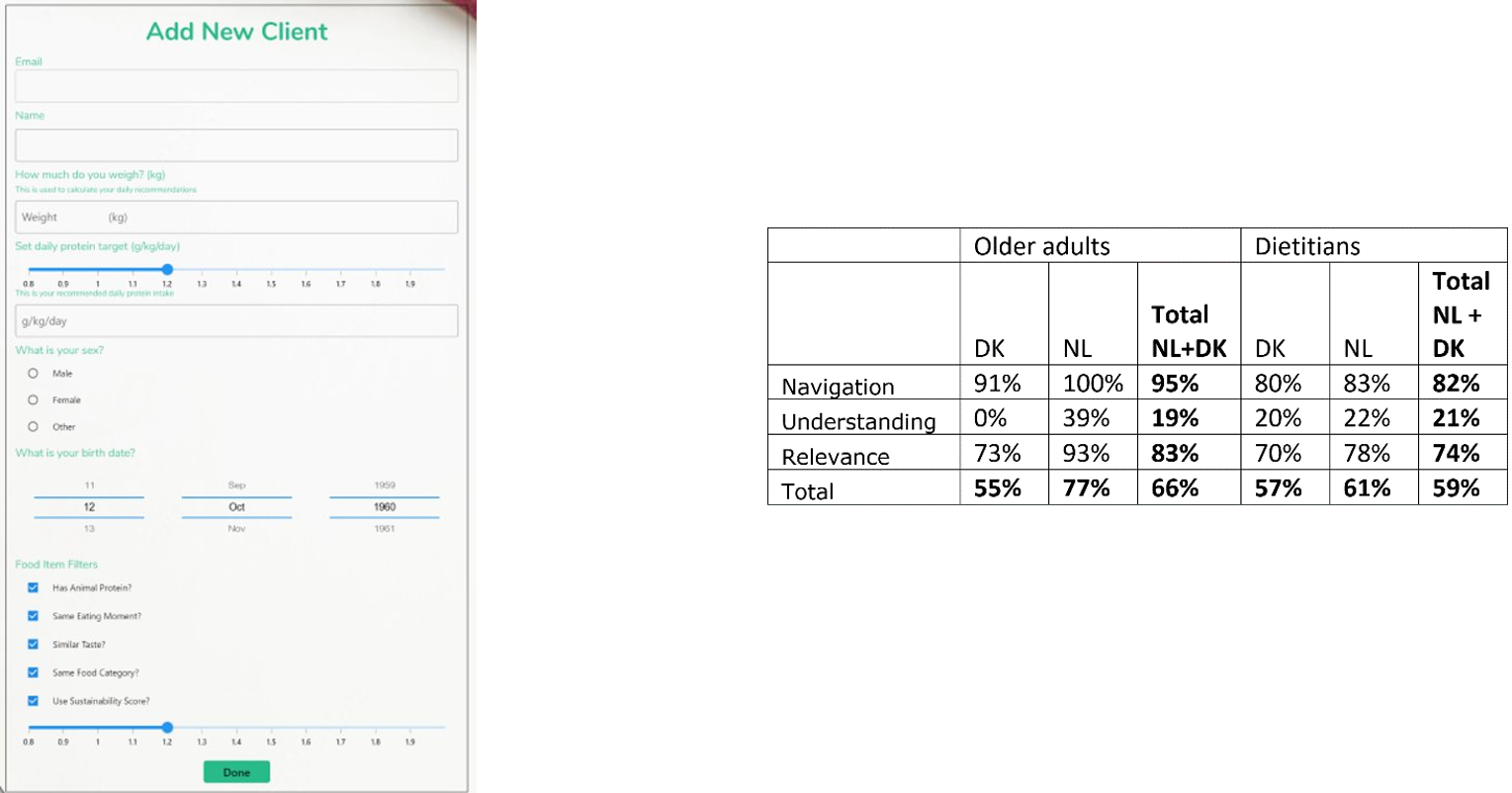


|  | Older adults | | | Dietitians | | |
| --- | --- | --- | --- | --- | --- | --- |
|  | DK | NL | **Total** | DK | NL | **Total** |
| Navigation | 91% | 100% | **95%** | 80% | 83% | **82%** |
| Understanding | 0% | 39% | **19%** | 20% | 22% | **21%** |
| Relevance | 73% | 93% | **83%** | 70% | 78% | **74%** |
| **Total** | **55%** | **77%** | **66%** | **57%** | **61%** | **59%** |

Screen 3A: Add Food Intake


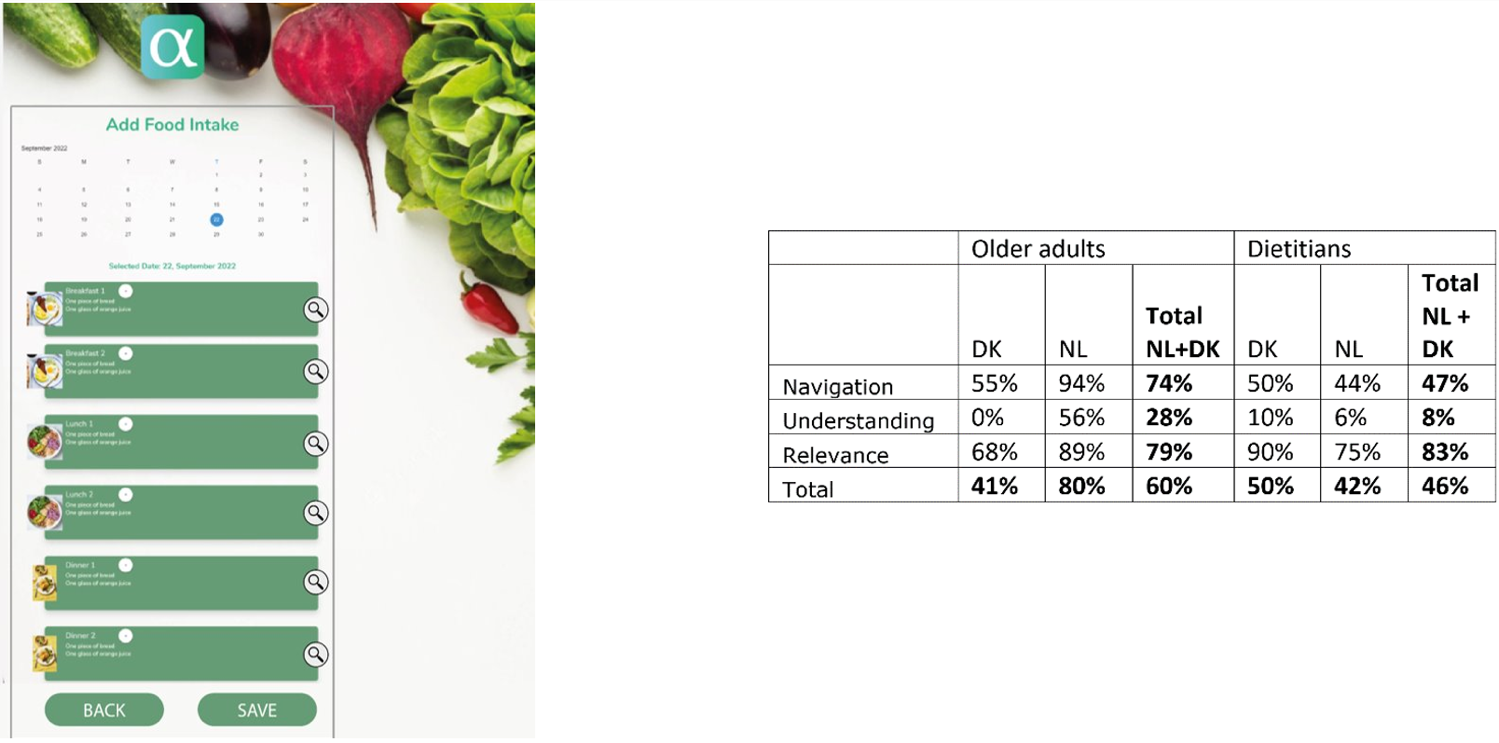


|  | Older adults | | | Dietitians | | |
| --- | --- | --- | --- | --- | --- | --- |
|  | DK | NL | **Total** | DK | NL | **Total** |
| Navigation | 55% | 94% | **74%** | 50% | 44% | **47%** |
| Understanding | 0% | 56% | **28%** | 10% | 6% | **8%** |
| Relevance | 68% | 89% | **79%** | 90% | 75% | **83%** |
| **Total** | **41%** | **80%** | **60%** | **50%** | **42%** | **46%** |

Screen 3B: Add Food Intake


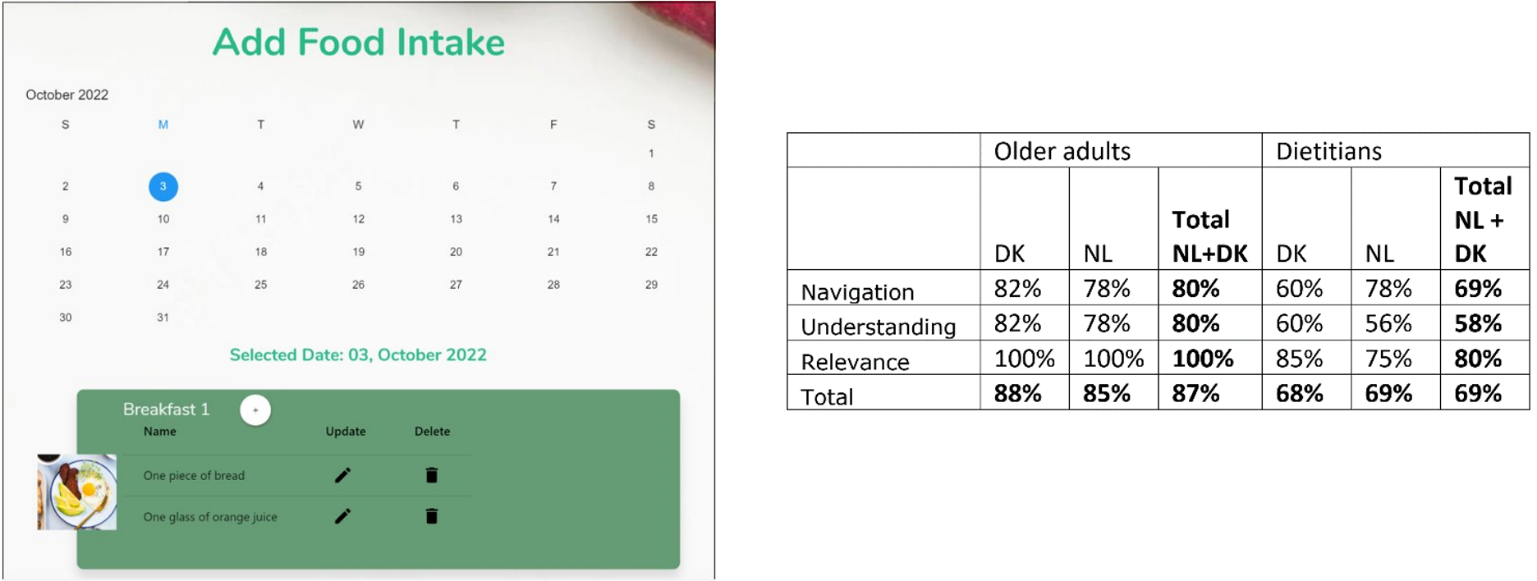


|  | Older adults | | | Dietitians | | |
| --- | --- | --- | --- | --- | --- | --- |
|  | DK | NL | **Total** | DK | NL | **Total** |
| Navigation | 82% | 78% | **80%** | 60% | 78% | **69%** |
| Understanding | 82% | 78% | **80%** | 60% | 56% | **58%** |
| Relevance | 100% | 100% | **100%** | 85% | 75% | **80%** |
| **Total** | **88%** | **85%** | **87%** | **68%** | **69%** | **69%** |

Screen 3C: Add Food Intake


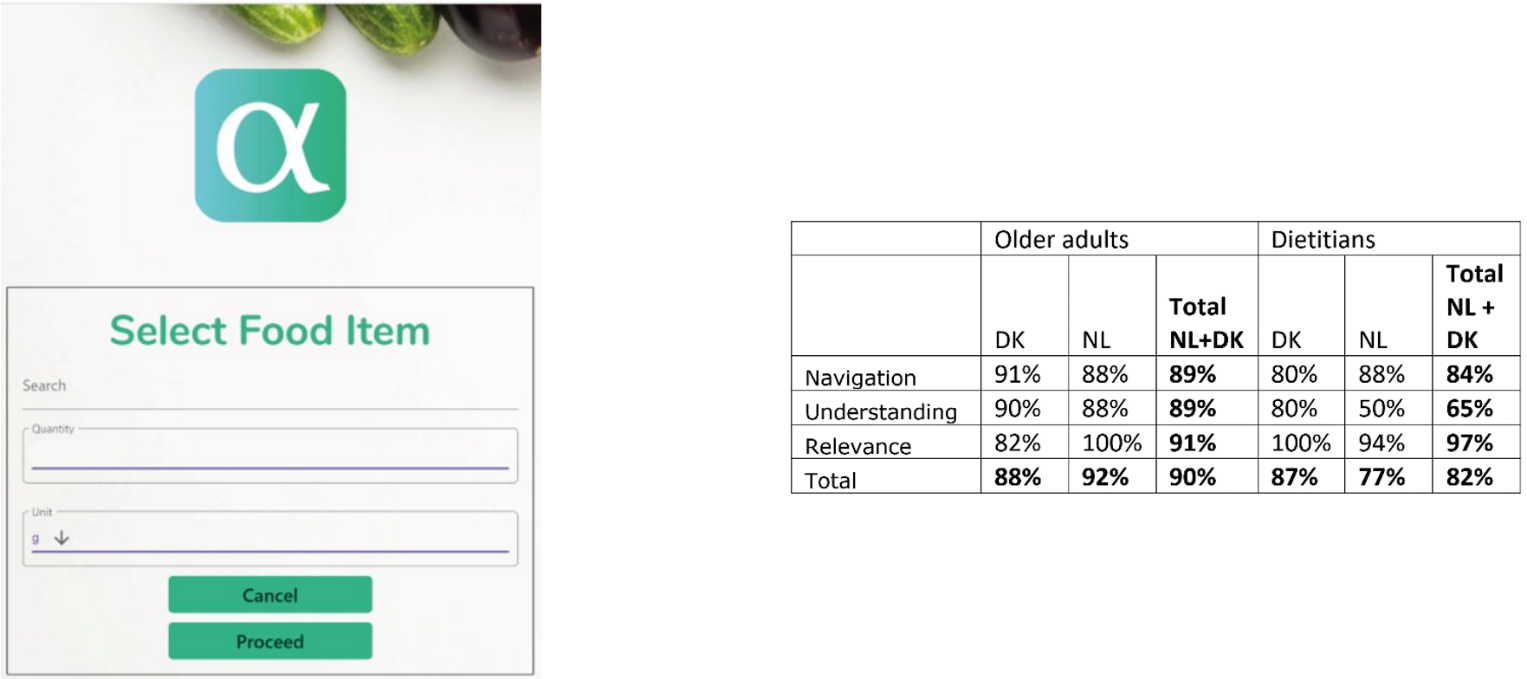


|  | Older adults | | | Dietitians | | |
| --- | --- | --- | --- | --- | --- | --- |
|  | DK | NL | **Total** | DK | NL | **Total** |
| Navigation | 91% | 88% | **89%** | 80% | 88% | **84%** |
| Understanding | 90% | 88% | **89%** | 80% | 50% | **65%** |
| Relevance | 82% | 100% | **91%** | 100% | 94% | **97%** |
| **Total** | **88%** | **92%** | **90%** | **87%** | **77%** | **82%** |

Screen 4A: New Consultation Session


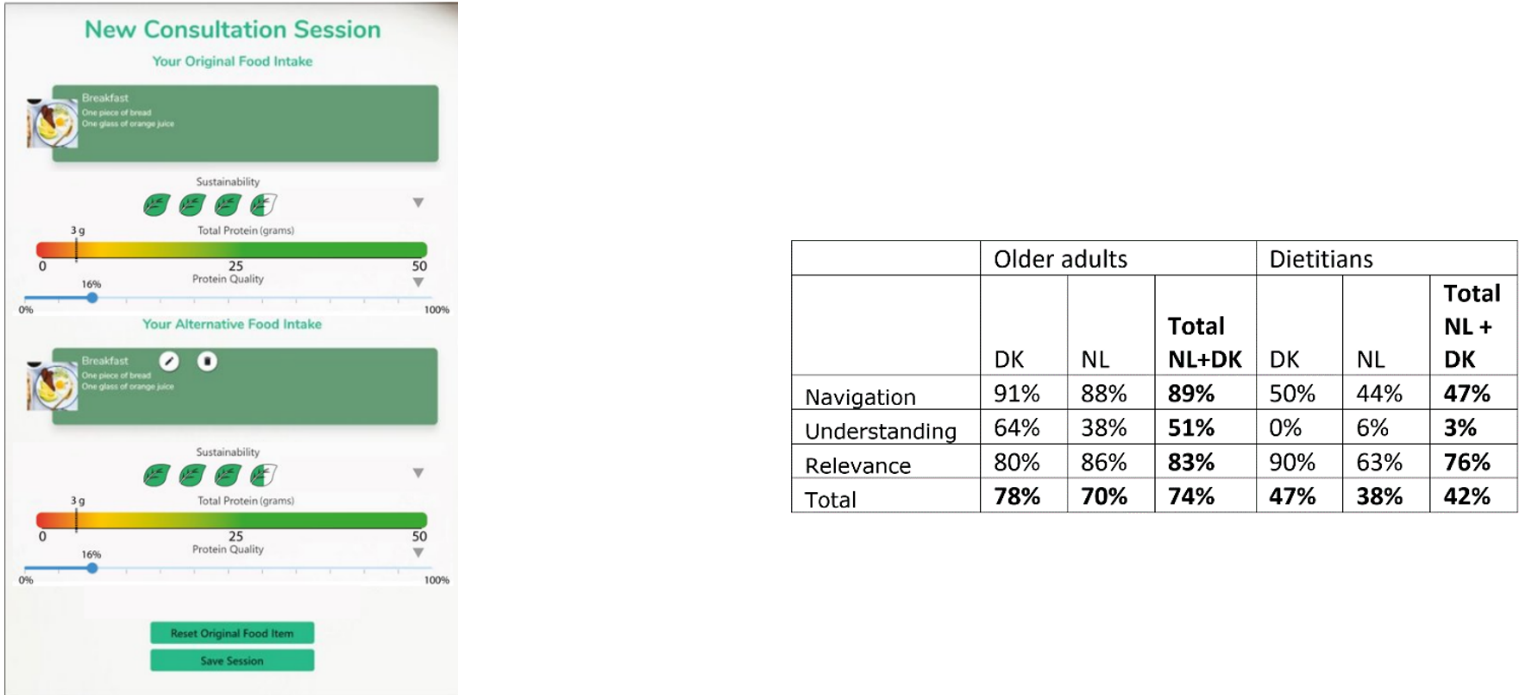


|  | Older adults | | | Dietitians | | |
| --- | --- | --- | --- | --- | --- | --- |
|  | DK | NL | **Total** | DK | NL | **Total** |
| Navigation | 91% | 88% | **89%** | 50% | 44% | **47%** |
| Understanding | 64% | 38% | **51%** | 0% | 6% | **3%** |
| Relevance | 80% | 86% | **83%** | 90% | 63% | **76%** |
| **Total** | **78%** | **70%** | **74%** | **47%** | **38%** | **42%** |

Screen 4B: New Consultation Session


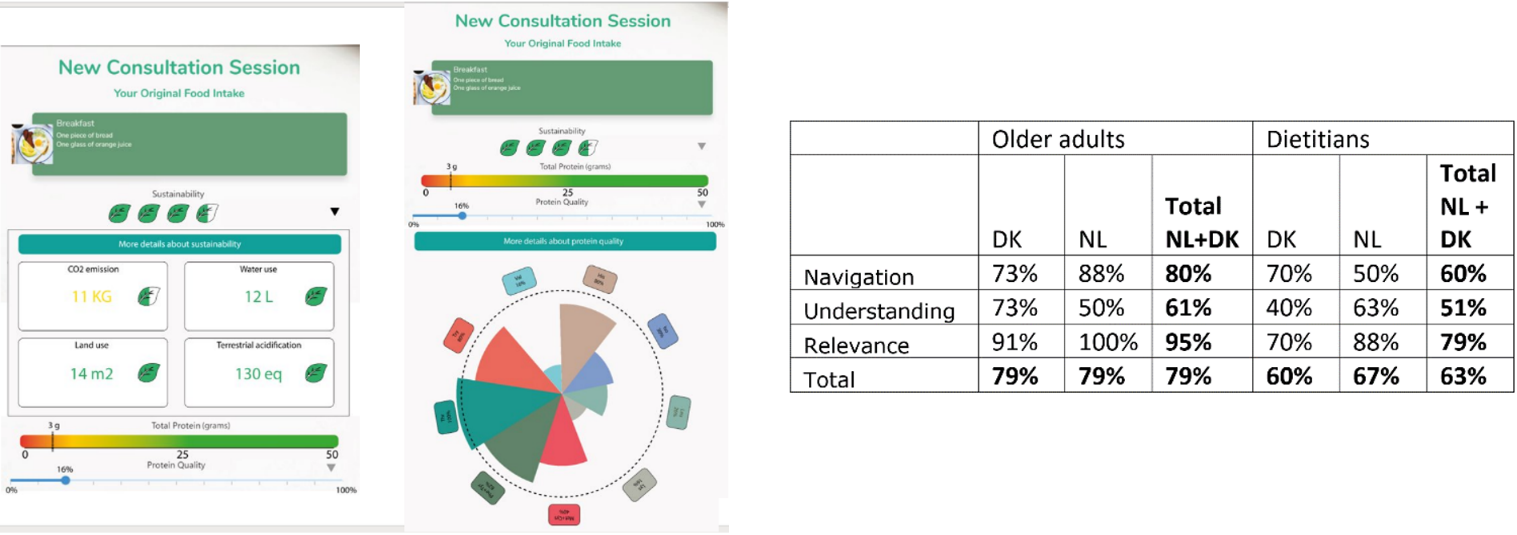


|  | Older adults | | | Dietitians | | |
| --- | --- | --- | --- | --- | --- | --- |
|  | DK | NL | **Total** | DK | NL | **Total** |
| Navigation | 73% | 88% | **80%** | 70% | 50% | **60%** |
| Understanding | 73% | 50% | **61%** | 40% | 63% | **51%** |
| Relevance | 91% | 100% | **95%** | 70% | 88% | **79%** |
| **Total** | **79%** | **79%** | **79%** | **60%** | **67%** | **63%** |

Screen 5: Alternatives


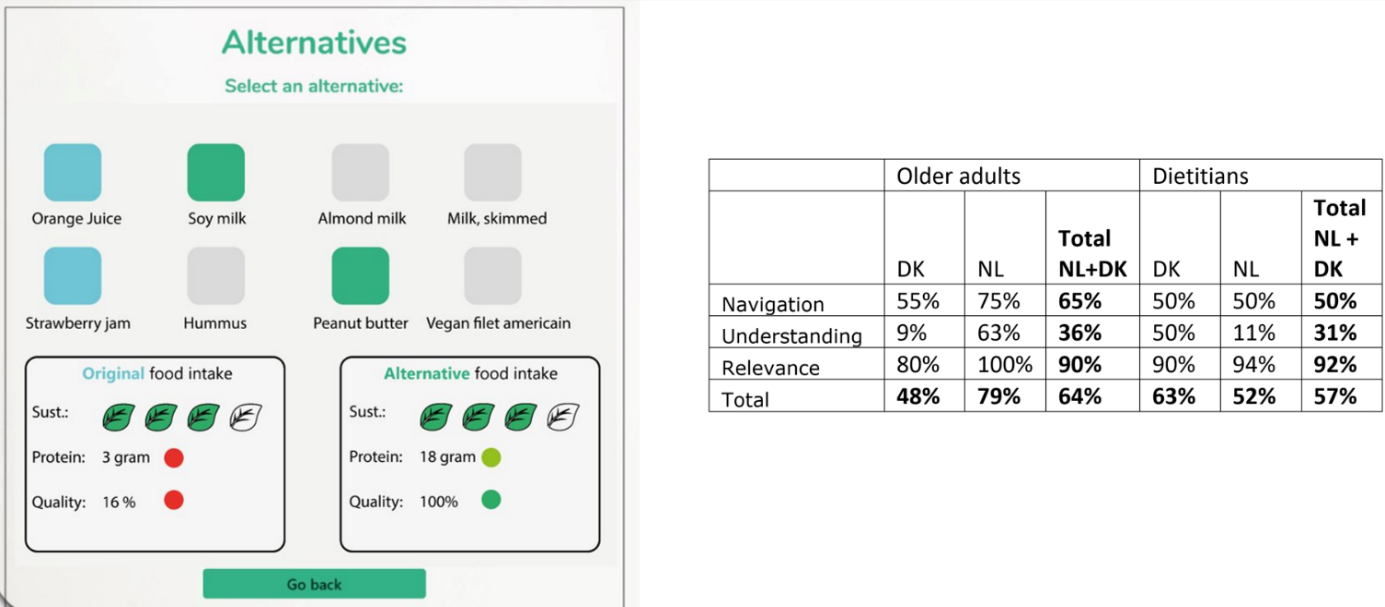


|  | Older adults | | | Dietitians | | |
| --- | --- | --- | --- | --- | --- | --- |
|  | DK | NL | **Total** | DK | NL | **Total** |
| Navigation | 55% | 75% | **65%** | 50% | 50% | **50%** |
| Understanding | 9% | 63% | **36%** | 50% | 11% | **31%** |
| Relevance | 80% | 100% | **90%** | 90% | 94% | **92%** |
| **Total** | **48%** | **79%** | **64%** | **63%** | **52%** | **57%** |
